# Bridging the social and the biomedical: engaging the social and political sciences in HIV research

**DOI:** 10.1186/1758-2652-14-S2-S1

**Published:** 2011-09-27

**Authors:** Susan C Kippax, Martin Holt, Samuel R Friedman

**Affiliations:** 1 Social Policy Research Centre, The University of New South Wales, Sydney, Australia; 2 National Centre in HIV Social Research, The University of New South Wales, Sydney, Australia; 3 National Development Research Institutes, New York, USA

## Abstract

This supplement to the *Journal of the International AIDS Society* focuses on the engagement of the social and political sciences within HIV research and, in particular, maintaining a productive relationship between social and biomedical perspectives on HIV. It responds to a number of concerns raised primarily by social scientists, but also recognized as important by biomedical and public health researchers. These concerns include how best to understand the impact of medical technologies (such as HIV treatments, HIV testing, viral load testing, male circumcision, microbicides, and pre-and post-exposure prophylaxis) on sexual cultures, drug practices, relationships and social networks in different cultural, economic and political contexts. The supplement is also concerned with how we might examine the relationship between HIV prevention and treatment, understand the social and political mobilization required to tackle HIV, and sustain the range of disciplinary approaches needed to inform and guide responses to the global pandemic. The six articles included in the supplement demonstrate the value of fostering high quality social and political research to inform, guide and challenge our collaborative responses to HIV/AIDS.

## 

HIV is a profoundly social disease, and its causes and consequences are deeply embedded in social, cultural and political processes. As noted in two reports for the International AIDS Society [1,2] and a number of papers [3-5], HIV has always had social, as well as biomedical, significance. The social sciences continue to play a central role in responses to HIV. Here we use the term “social science” to include a range of disciplines, such as anthropology, cultural studies, economics, geography, international relations, political science, social psychology and sociology.

The different profiles of HIV epidemics, generalized and concentrated, underscore the central role played by social, cultural and political factors in the transmission of HIV. For example, it would be hard to understand the generalized, heterosexually driven epidemics within many African countries without reference to gender inequality, poverty and an unstable health infrastructure in many settings. In contrast, concentrated epidemics among people who inject drugs or men who have sex with men are driven by stigmatized practices (sharing injecting equipment, unprotected anal intercourse), and responses to those epidemics can be hampered by punitive laws and a lack of political will to provide harm-reduction measures (such as needle exchange and condoms) [6].

The responses of individuals, communities and governments to epidemics vary dramatically - again as an expression of, and shaped by, social processes. In many countries and regions, HIV has caused fear and discrimination, while in others, it has triggered responses of solidarity and community activism. The impact of HIV and AIDS on individuals, households and communities, as well as on nations and regions, also varies, with HIV and AIDS affecting the socio-economic, cultural and political fabric of countries and regions.

Notwithstanding the importance of social science, an increasing tendency to neglect the social sciences in HIV prevention, treatment and care has been noted, following what might be regarded as an intense period of “bio-medicalization” of the HIV response [1,7]. This is a cause for concern as the social sciences are essential to complement, strengthen and situate biomedical research, as well as independent fields that can identify additional ways forward in the global pandemic. Maintaining a critical perspective on developments within the HIV field is important, but is often a risky endeavour in a field dominated by biomedical research. Alternatively, collaboration between the social and biomedical sciences, seen by many as essential for progress within the epidemic, can be a complex and testing process [1].

This supplement of the *Journal of the International AIDS Society* focuses on the engagement of the social and political sciences within HIV research and, in particular, maintaining a productive relationship between social and biomedical perspectives on HIV. It responds to a number of concerns raised primarily by social scientists, but also recognized as important by biomedical and public health researchers. These concerns include how best to understand the impact of medical technologies (such as HIV treatments, HIV testing, viral load testing, male circumcision, microbicides, and pre- and post-exposure prophylaxis) on sexual cultures, drug practices, relationships and social networks in different cultural, economic and political contexts. The supplement is also concerned with how we might examine the relationship between HIV prevention and treatment, understand the social and political mobilization required to tackle HIV, or sustain the range of disciplinary approaches needed to inform and guide responses to the global pandemic.

We had an overwhelming response to the announcement of the supplement: more than 150 abstracts were submitted for consideration. After reviewing these submissions, we invited 10 authors to prepare full papers for peer review. The six articles presented here successfully completed the peer review process and, we believe, make for stimulating reading.

Using the example of “treatment as prevention” Barry Adam considers how we might overcome the tensions between biomedical and social approaches to HIV prevention [8]. He argues for a robust social science research agenda that focuses on locally embedded practices, in contradistinction to biomedical approaches that offer technological developments without reference to social and community needs. Adam makes the pointed observation that any intervention in the epidemic, whether it is understood as “biomedical”, “behavioural” or both, requires community engagement and mobilization in order to stand any chance of success.

A number of contributors take up the challenge of understanding how local needs do or don’t mesh with the aims of biomedical research with reference to large, international trials of biomedical HIV prevention technologies. Kathleen MacQueen reflects on the challenges of integrating social, behavioural, biomedical and ethical perspectives based on her long engagement in biomedical HIV prevention trials [9]. She notes that “[s]ocial scientists are now integrated as members of biomedical HIV prevention trial research teams, yet social science is minimally integrated with the science of biomedical HIV prevention”. MacQueen’s paper reminds us that social scientists working in HIV research often feel that they have no choice but to either adapt to the priorities of biomedicine and public health or maintain an autonomous HIV social science agenda outside of biomedical research [1]. From MacQueen’s perspective, such an opposition is insufficient to effectively enfold social science within biomedical prevention trials, and she argues for closer collaboration in trial design, despite the potential tensions.

Catherine Montgomery and Robert Pool offer an example of social scientists engaging in biomedical prevention trials, with reference to their experience on the Microbicides Development Programme (MDP) 301 trial of the microbicide candidate PRO 2000 [10]. They describe how anthropological research conducted throughout the trial revealed that trial participants often understood and made use of the microbicide gel in ways that were completely unanticipated by trial researchers. However, despite recognition that social science methods generated valuable insights into the conduct and outcomes of the trial, the existing hierarchy of evidence within the randomized controlled design meant that these findings had limited impact on the conduct of the trial itself. Despite the difficulties in reconciling different epistemologies and versions of evidence, Montgomery and Pool conclude that the well-funded integration of social science within the MDP 301 trial demonstrates the advantages of social and biomedical researchers working together and is an approach that should be pursued and maintained.

The other contributors to the supplement consider the political, organizational and structural aspects of HIV programmes and how these aspects affect the outcomes of HIV programmes. The paper by Ashley Fox, Allison Goldberg, Radhika Gore and Till Bärnighausen critically reviews efforts to conceptualize political commitment in HIV responses and the linkages between political commitment and “success” in those responses, such as declines in HIV infection rates and AIDS-related mortality [11]. The paper addresses what political commitment means across a number of dimensions, and suggests how it should be assessed in resource-limited and resource-rich settings. We believe the contribution of Fox and her colleagues responds to calls to further develop conceptual tools to frame and understand country responses to HIV.

Rachel Robinson has a similar goal: to understand why some countries appear to respond more effectively to HIV than others [12]. In contrast to Fox and colleagues’ focus on political commitment, Robinson studies a number of organizational and structural determinants of HIV outcomes, analyzing the historical development of family planning and reproductive health services in sub-Saharan Africa. Robinson shows that countries with the greatest declines in HIV prevalence and incidence were significantly more likely to have well-established family planning and reproductive health service networks. She also finds that epidemiological outcomes are associated with population policies, relative wealth, cultural diversity and colonial history. The findings other study suggest that family planning organizations should be strengthened to assist in country responses to HIV, but that this type of “structural intervention” may take many years to become well established.

Kathrin Frey and Daniel Kübler shed light on the difficulties of sustaining HIV social science research and multidisciplinary approaches to HIV in their analysis of funding policies in Switzerland [13]. They describe the shift from a dedicated funding mechanism for HIV social science research to a model in which HIV social scientists apply and compete for funding through a national, generalized peer review model. The result has been a dramatic reduction in the number of HIV social science research projects developed and funded in Switzerland. Many readers will have observed similar shifts in their own countries and regions as the push continues to “normalize” HIV’s place in public health responses and funding mechanisms. Whether there is a need for specialized social science funding programmes within the global HIV epidemic, and whether such funding may need to be considered “normal” for many other diseases, is a debate that is sure to continue.

The *Journal of the International AIDS Society* is pleased to launch this supplement. We believe the contributors to this supplement demonstrate the value of fostering high quality social and political research to inform, guide and challenge our collaborative responses to HIV/AIDS. By supporting the publication of this supplement, the International AIDS Society underlines its commitment to social science research, which it fosters through a range of activities, including this journal and international conferences. We hope the issues and debates raised here will engage a broader audience, including community members, clinicians, policymakers and academics. We would like to encourage readers to consider the implications of these debates for their and others’ HIV-related research and to maintain dialogue on the entangled, intimate and productive relationships between the social and the biomedical.

## Competing interests

The authors declare that they have no competing interests.
